# Optimal specific wavelength for maximum thrust production in undulatory propulsion

**DOI:** 10.1371/journal.pone.0179727

**Published:** 2017-06-27

**Authors:** Nishant Nangia, Rahul Bale, Nelson Chen, Yohanna Hanna, Neelesh A. Patankar

**Affiliations:** 1 Engineering Sciences and Applied Mathematics, Northwestern University, Evanston, IL, United States of America; 2 Department of Mechanical Engineering, Northwestern University, Evanston, IL, United States of America; University at Buffalo - The State University of New York, UNITED STATES

## Abstract

What wavelengths do undulatory swimmers use during propulsion? In this work we find that a wide range of body/caudal fin (BCF) swimmers, from larval zebrafish and herring to fully–grown eels, use specific wavelength (ratio of wavelength to tail amplitude of undulation) values that fall within a relatively narrow range. The possible emergence of this constraint is interrogated using numerical simulations of fluid–structure interaction. Based on these, it was found that there is an optimal specific wavelength (OSW) that maximizes the swimming speed and thrust generated by an undulatory swimmer. The observed values of specific wavelength for BCF animals are relatively close to this OSW. The mechanisms underlying the maximum propulsive thrust for BCF swimmers are quantified and are found to be consistent with the mechanisms hypothesized in prior work. The adherence to an optimal value of specific wavelength in most natural hydrodynamic propulsors gives rise to empirical design criteria for man–made propulsors.

## Introduction

Dimensionless quantities are used to directly compare the biomechanics between systems of different scales [1]. The Strouhal number (*St* = 2*fa*/*U*, where *f* is the tail or wing beating frequency, 2*a* is the maximum tip–to–tip lateral excursion, and *U* is the forward speed) is a nondimensional number used to describe the kinematics of flying and swimming animals [2, 3]. Although it has been shown that some species of body/caudal fin (BCF) swimmers cruise at 0.2 < St < 0.4, there is evidence to support that the Strouhal number varies over a larger range of 0.6 − 2.2 for anguilliform and low Reynolds number undulatory swimmers [4, 5]. It is suggested that natural selection drives animals to this range of Strouhal numbers because it maximizes propulsive efficiency (ratio of hydrodynamic power output to mechanical power input) during cruising [2, 6]. Another dimensionless number called the specific wavelength (SW), the ratio of wavelength λ to an amplitude length scale, has been used to describe the kinematics of elongated fin (EF) swimmers [7]. Furthermore, SW has been shown through simulations and experiments to maximize net propulsive force for EF swimmers when it achieves a value near 20. This has been referred to as the optimal specific wavelength or OSW [7].

EF swimmers are characterized by a flexible elongated fin that runs lengthwise with the rigid body, allowing greater maneuverability but at slow speeds. The amplitude of the undulations vary along the length and span of the fin. On the other hand, BCF fish undulate their bodies and caudal fin to produce greater thrust and accelerations [1, 8]. The amplitude for BCF swimmers generally varies along the length, but not the span of the swimmer (although the amplitude can vary along the span of the caudal fin [9]). Additionally, the tail amplitude *a* is crucial in producing propulsive thrust [10], and therefore the relevant specific wavelength for BCF swimmers is SW = λ/*a*. It seems likely that BCF swimmers would benefit from adherence to OSW because it ensures that speed can be maximized given a set of kinematic parameters irrespective of whether the animal swims efficiently during cruising or less efficiently during attack or evasive maneuvers. Here we show that undulatory BCF swimmers also abide to a relatively narrow range of SW. For this type of propulsion, the observed steady–swimming kinematics are found to optimize net axial force over a wide range of length and velocity scales.

In this work, a meta–analysis was done using data from 27 species of undulatory BCF swimmers to find typical Strouhal numbers and specific wavelengths observed in these animals. Previous analyses of these data showed that the observed Strouhal number depends nonlinearly on the Reynolds number (Re = *ρUL*/*μ*, where *U* is the fish’s forward swimming speed, *L* is the fish’s length, and *ρ* and *μ* are the density and viscosity of water, respectively) [4]. Reanalyzing these data, we found that swimming mode and aspect ratio (AR) play a role in the variation of St. Additionally, a numerical study done in this work is used to establish an optimality condition relating net axial force and specific wavelength of BCF swimmers. The meta–analysis and simulation data were compared to OSW measurements obtained from parametric studies done on robotic undulating sheets [11, 12]. Based on these analyses, we find reasonable adherence by BCF swimmers to the OSW, and a mechanical reason for the possible emergence of this phenomenon.

## Results

### Meta–analysis

We first investigated whether or not BCF animals swim near a constant specific wavelength value. Since the most relevant amplitude length scale for body/caudal fin swimmers is the tail amplitude [10], the specific wavelength is defined to be λ/*a*. Specific wavelength was calculated for 27 species (28 groups since distinction is made between young and adult axolotl) of steady–swimming BCF swimmers based on data from a meta–analysis study [4]. These observations cover a wide range of lengths (0.3 cm for larval zebrafish to 69.5 cm for Atlantic cod), velocities (0.249 *L*/s for longnose gar to 54.0 *L*/s for larval Atlantic herring), and swimming modes (anguilliform to thunniform [8]). These data also include observations from adult and larval Mexican salamanders, which swim with an anguilliform motion [13, 14]. The lateral Reynolds number (Re_lat_ = *ρ*(*fa*)*a*/*μ*, where *a* and *f* are the undulation tail amplitude and frequency, respectively) was calculated for all of these observations and ranged from 2.55 × 10^1^ to 8.55 × 10^3^. The corresponding range in swimming–speed based Reynolds number was 2.10 × 10^2^ to 7.71 × 10^5^. For the swimming animals considered in this study from [4], the relationship between Re and Re_lat_ is shown in section 1 of S1 Appendix. We chose to analyze this data using Re_lat_ instead of Re to allow for a direct comparison to our translation–locked fin simulations, for which there is no measured swimming speed (see Parametric Study). Additionally for free–swimming simulations, Re_lat_ is a parameter that is known *a priori* and can be prescribed at the beginning of the simulation. On the other hand, Re is an output of the simulation, which is not preferable when conducting parametric studies.

For the elongated fin swimmers considered in [7], it was found that the ratio of wavelength to average amplitude *ã* was near 20 for a wide range of aquatic invertebrates and vertebrates. Since the amplitude *a* at the tail tip was chosen to define specific wavelength in this study and we observed that *a*/*ã* was between 1.53 and 2.56 for these body/caudal fin swimmers, we chose to interrogate whether or not these animals undulate at SW values near 10. Letting *x*_*ij*_ denote observation *j* from group *i*, we calculated mean quantities for group *i* with *N*_*i*_ observations as ${\bar{x}}_{i}=\frac{1}{N_{i}}\sum_{j=1}^{N_{i}}\;x_{ij}$, and overall averages $\bar{x}=\frac{1}{28}\sum_{i=1}^{28}\;{\bar{x}}_{i}$. We also calculated the overall standard deviations as $s_{x}=\sqrt{\frac{1}{27}\;\overset{28}{\underset{i=1}{\sum }}\;{\left({\bar{x}}_{i}-\bar{x}\right)}^{2}}$. We found that these fish species swim at specific wavelengths between 4.02 and 14.93 with a mean value of $\bar{\text{SW}}=9.91$ and standard deviation *s*_SW_ = 3.45. We performed a two–sided *t*–test on the average specific wavelength ${\bar{\text{SW}}}_{i}$ for the *n* = 28 groups of swimmers. Although the data are not normally distributed, we chose the *t*–test because of its robustness to large deviations from the normality assumption. For an *α*–level of 0.05, we found that the average $\bar{\text{SW}}=9.91$ did not vary significantly (*P* = 0.888) from 10, with a 95% confidence interval (CI) of $8.57<{\bar{\text{SW}}}_{i}<11.25$. The median ${\bar{\text{SW}}}_{i}$ is 10.30.

**Fig 1:**
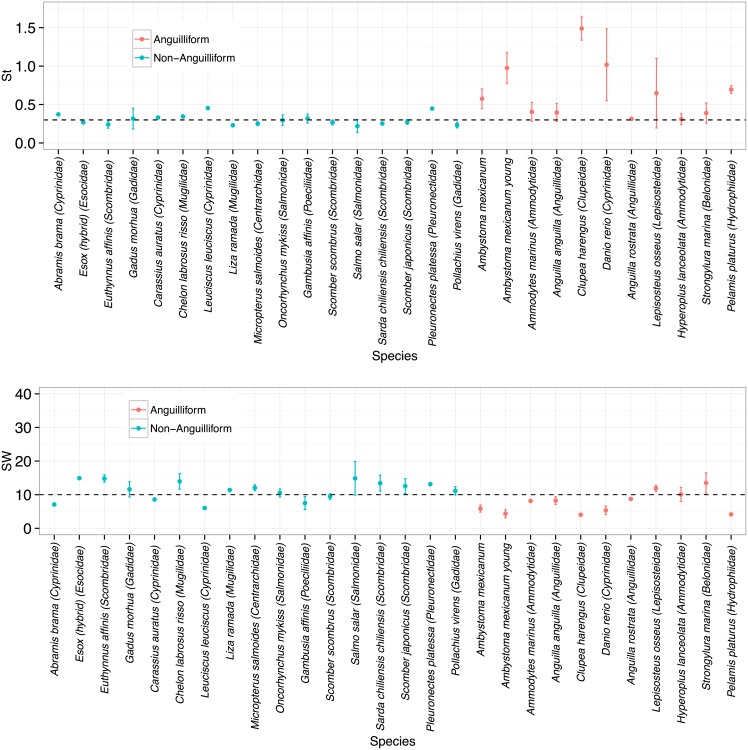
Observed St and SW ranges from meta–analysis data of body/caudal fin swimmers. The Strouhal number (top) and specific wavelength (bottom) for the species considered in this study. Data points represent average values, ${\bar{x}}_{i}$ where *x* ∈ {St, SW}, of individual species and error bars indicate ± one s.d. from the species mean. Dashed lines represent St = 0.3 and SW = 10. Distinction is made between anguilliform and non–anguilliform swimmers. Data labeled as *Ambystoma mexicanum* and *Ambystoma mexicanum young* are from adult and larval axolotl, respectively. All observations of *Clupea harengus* and *Danio rerio* came from anguilliform larvae. For some species, the error bars are not visible at this scale, while for others, only one observation is recorded and no error bars are available; see Table 1 for more details. The data are available in S1 Data.

Fig 1 shows the mean St and SW for each of these 28 groups. It is seen that the range of Strouhal numbers depends on the mode of swimming; St is clustered around 0.3 for non–anguilliform (subcarangiform, carangiform, or thunniform) swimmers while St consistently overshoots 0.3 for anguilliform swimmers. The mean Strouhal number for non–anguilliform swimmers is 0.30, while for anguilliform swimmers it is 0.66. For anguilliform swimmers, this average varied significantly from 0.3 (*n* = 11, *P* = 0.01, 95% CI: $0.41<{\bar{\text{St}}}_{i}<0.90$).

**Fig 2:**
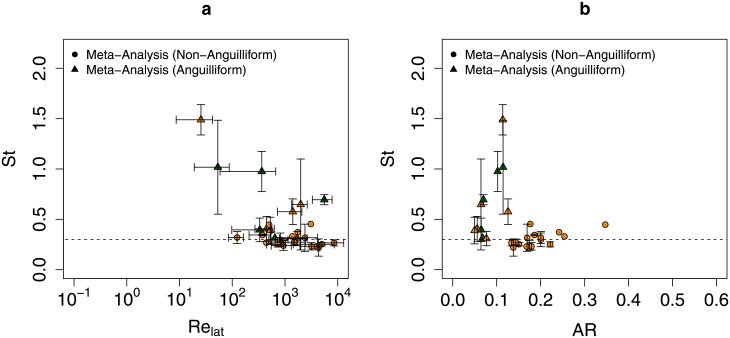
Variability in St as a function of Re_lat_ and AR. Intraspecies mean Strouhal number vs. (a) lateral Reynolds number, and (b) aspect ratio for observed non-anguilliform(⚫) and anguilliform(▲) swimmers. Of these, orange (green) points represent swimmers with (without) well–defined caudal fins. Error bars indicate ± one s.d. from the species mean. For some species, the error bars are not visible at this scale, while for others, only one observation is recorded and no error bars are available; see Table 1 for more details. Data are available in S1 Data and Table 1.

It is seen that the observed variation in SW is no more than that in St; SW did not vary as much as St with respect to different swimming modes. Are there traits common to these anguilliform swimmers that correlate to higher Strouhal number locomotion? To answer this, we plotted St vs. Re_lat_ and St vs. AR in Fig 2 for both anguilliform and non-anguilliform swimmers. We found that non-anguilliform swimmers were clustered around St = 0.3 and their aspect ratios tend to be higher (AR > 0.133; Fig 2b). Here the aspect ratio is AR = (area of fish body)/(length of fish body)^2^. Anguilliform swimmers with similar lateral Reynolds numbers as non-anguilliform swimmers have in general higher values of St. This trend may be due to the low aspect ratios of anguilliform swimmers, which tend to be longer and skinnier and swim at higher St values (Fig 2b). Additionally, anguilliform swimmers at low Reynolds numbers tend to have even higher values of St as seen in previous studies [4, 15]. For example, notice that the two larval anguilliform species that swim at Re_lat_ < 10^2^ (*Danio rerio* and *Clupea harengus*) also swim at St > 1. Thus, we hypothesize that low aspect ratios cause higher St for anguilliform swimmers and low Re swimming tends to further increase the observed values of St in anguilliform swimmers.

**Fig 3:**
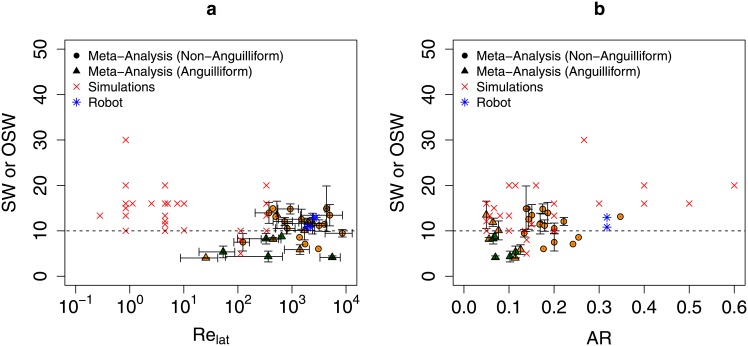
Variability in SW and OSW as a function of Re_lat_ and AR. Intraspecies mean specific wavelength vs. (a) lateral Reynolds number, (b) aspect ratio for observed non-anguilliform(⚫) and anguilliform(▲) swimmers. Of these, orange (green) points represent swimmers with (without) well–defined caudal fins. Red crosses (×) represent the optimal specific wavelength for simulations done in the present study. Blue asterisks (*) represent the optimal specific wavelengths for robotic undulating sheets reported in [11, 12]. Error bars indicate ± one s.d. from the species mean. For some species, the error bars are not visible at this scale, while for others, only one observation is recorded and no error bars are available; see Table 1 for more details. Data are available in S1 Data and Table 1.

Finally Fig 3 shows SW as a function of Re_lat_ and AR for these species. We did not see a variation pattern in SW due to either of these parameters as was seen with St. Body/caudal fin swimmers were found to adhere to a similar SW constraint as elongated fin swimmers [7]. A possible physical basis of this constraint in BCF swimmers is explored in the next section using numerical simulations.

### Numerical simulations

#### Nondimensionalization

Motivation for the present numerical study came from dimensional analysis. Consider a rectangular plate immersed in a fluid with a prescribed traveling wave undulation pattern given by,

$$y\left(x,t\right)=a\sin\;(\frac{2\pi }{\lambda }\left(x-\lambda ft\right)),$$ (1)

in which *f* is the frequency of the traveling wave, λ is the wavelength, and *a* is the amplitude of the undulation, which is taken to be constant. Assuming that a steady periodic flow state has been reached and that the translational degrees of freedom of the sheet have been locked, the net static axial propulsive force *F*_*x*_ depends on the following physical parameters:

$$F_{x}=fn\left(\rho ,\mu ,f,\lambda ,L,h,a\right),$$ (2)

in which *fn* denotes “function of,” *ρ* is the fluid density, *μ* is the fluid viscosity, and *L* and *h* are the length and span of the plate, respectively. Note that since there is no far–field velocity opposing the plate and the plate is locked in place, there is no velocity present in Eq 2.

Using the Buckingham Pi Theorem, Eq 2 can be nondimensionalized as,

$$\hat{F}=\frac{F_{x}}{\frac{1}{2}\rho {\left(fa\right)}^{2}Lh}=fn\;(\frac{\lambda }{a},\frac{\rho (fa)a}{\mu },\frac{h}{L},\frac{h}{a})=fn\;(\text{SW},{\text{Re}}_{\text{lat}},\text{AR},\frac{h}{a}).$$ (3)

Here, the dimensionless force $\hat{F}$ depends on specific wavelength SW, lateral Reynolds number Re_lat_, plate aspect ratio AR = *Lh*/*L*^2^ = *h*/*L*, and a scaled height parameter *h*/*a*. Reynolds numbers based on the wave–speed Re_wave_ = *ρ*(*λf*)*L*/*μ* or lateral speed and length Re_lat,*L*_ = *ρ*(*fa*)*L*/*μ* would have been equally valid choices in our nondimensionalization. We chose to include the lateral Reynolds number in Eq 3 because there was no swimming speed associated with Eqs 2 and 3, and this choice separates the dimensionless quantities depending on frequency and wavelength. For the swimming animals considered in this study, all of these Reynolds numbers are strongly correlated with each other and the swimming–speed Reynolds number Re (see section 1 of S1 Appendix). Hence, our analysis is independent of the chosen Reynolds number. In the present numerical study, simulations gave insight on what range of specific wavelength maximizes net propulsive force. We also explored how AR, Re_lat_, and *h*/*a* affected the optimal specific wavelength (OSW).

#### Parametric study

Using numerical simulations of small sheets and scaled–down eel and mackerel bodies, we interrogated whether an optimization principle exists between net axial force and specific wavelength for highly undulatory bodies.

Two types of simulations were carried out in this study: translation–locked and free–swimming. For translation–locked simulations, let **F** = (*F*_*x*_, *F*_*y*_, *F*_*z*_) be the transient net force felt on the swimmer in the *x*, *y*, and *z* directions and for free–swimming simulations, let **U** = (*U*_*x*_, *U*_*y*_, *U*_*z*_) be the center–of–mass (COM) velocity of the swimmer. In the translation–locked simulations, the undulating body was fixed in place and **F** was measured while in the free–swimming case, the body’s COM velocity **U** was solved for by the simulation and the body self–propelled. In both cases, the rotational degrees of freedom of the swimmer were locked. The simulations carried out in this study were “fully–resolved”, meaning that the fluid–solid coupling was not modeled using drag laws; the flow field, net propulsive force, swimming speed, and power generated by the swimmer were outputs of each simulation. Moreover, all the dynamics of the swimming system were captured in our simulations, including the effect of linear recoil. For translation–locked simulations, the recoil effect led to oscillations in the sway *F*_*y*_ and heave *F*_*z*_ forces. For free–swimming simulations, the recoil effect led to an oscillation of the swimmer’s center of mass in the sway *U*_*y*_ and heave *U*_*z*_ directions, which were outputs of the simulation. Both axial swimming speed and net axial force oscillate about a mean value in either case.

**Fig 4:**
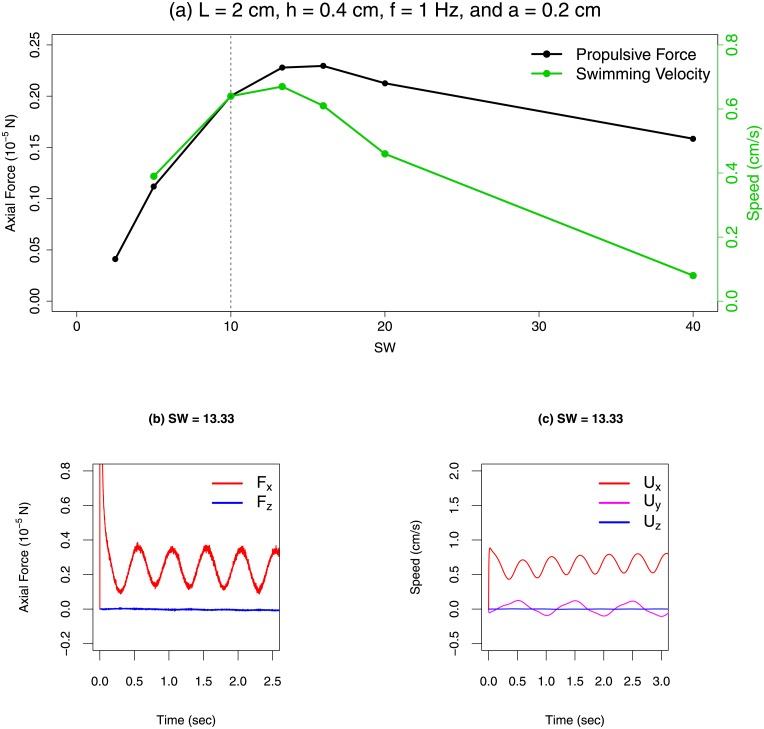
Measured swimming speed and force from undulating sheet simulations. (a) Axial swimming speed and propulsive force computed from free–swimming (green) and translation–locked (black) simulations of rectangular sheets plotted against the specific wavelength. In free–swimming simulations, the forward swimming speed of the undulating plate was an output parameter of the simulation. Simulations were carried out at a lateral Reynolds number Re_lat_ = 4.49, with corresponding swimming–speed Reynolds number range 1.8 × 10^1^ < Re < 1.51 × 10^2^. Data are available in S2 Data. (b) Evolution of axial *F*_*x*_ and heave forces *F*_*z*_ over time for a translation–locked, undulating sheet simulation with SW = 13.33. The oscillation in *F*_*z*_ is a signature of the linear recoil effect on the swimmer. The sway force *F*_*y*_ is not shown because the kinematics of the swimmer’s undulation lead to large *F*_*y*_ values, although it also oscillates about a mean value. (c) Evolution of **U** in each coordinate direction over time for a self–propelled, undulating sheet simulation with SW = 13.33. The oscillation of the heave velocity *U*_*z*_ about 0 is not easily visible at this scale.

Fig 4a shows the correlation between propulsive thrust *F*_*x*_ and swimming velocity *U*_*x*_ by comparing the results from the stationary and free–swimming plate simulations. We indeed see that both swimming speed and thrust are maximized by similar SW values. Additionally, *F*_*x*_ and *U*_*x*_ are strongly correlated for SW < 20 and their peaks occur at similar OSW values. For higher SW, their correlation is weaker, although both *F*_*x*_ and *U*_*x*_ decrease with increasing SW in this range. We note that all the observed animals in the meta–analysis swam at SW between 4 and 15. The transient behaviors of **F** and **U** are shown in Fig 4b and 4c. More details of the numerical simulation technique and parameters are provided in the Materials and Methods section. Hereafter, we focus on analyzing propulsive force from stationary simulations, although we expect that the swimming speed computed from self–propelling simulations would follow similar trends with respect to SW.

We carried out simulations of stationary rectangular small–sheet (0.5 − 8 × 0.1 − 1.2 cm^2^) and large–sheet (10 − 20 × 1 − 2 cm^2^) sinusoidally undulating bodies with various wavelengths (0.25 − 40 cm), amplitudes (0.05 − 1.0 cm), and frequencies (1 − 4 Hz). For fixed length, span, frequency, and amplitude, the wavelength was varied and the OSW was taken to be the value of λ/*a* that maximized the force in the axial direction. The length and lateral speed scales for our simulated sheets were chosen to match those of swimmers with available kinematic data. Small–sheet simulations had Re_lat_ ∼ 10^−1^ − 10^2^ similar to species like larval *Clupea harengus* and *Danio rerio*, while large–sheet simulations had Re_lat_ ∼ 10^2^ − 3 × 10^2^ similar to species like *Anguilla anguilla* and *Micropterus salmoides*. Since there was no swimming–speed associated with these simulations, Re = 0. The relationship between Re and Re_lat_ was calculated for swimming animals in [4] and is shown in section 1 of S1 Appendix. We used aspect ratios ranging from 0.05 to 0.6, which is the AR range found for the BCF swimmers considered in this study.

**Fig 5:**
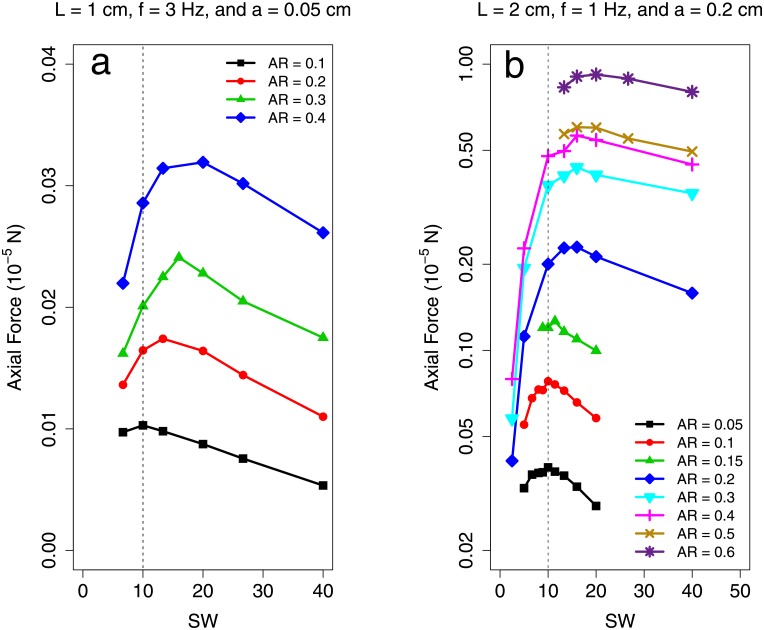
Measured propulsive force from low Re_lat_ undulating sheet simulations. The axial propulsive force generated by a stationary undulating sheet plotted against specific wavelength. In both cases (a) & (b), plate span was varied. These data represent cases where Re_lat_ < 1 × 10^2^. Data are available in S2 Data.

**Fig 6:**
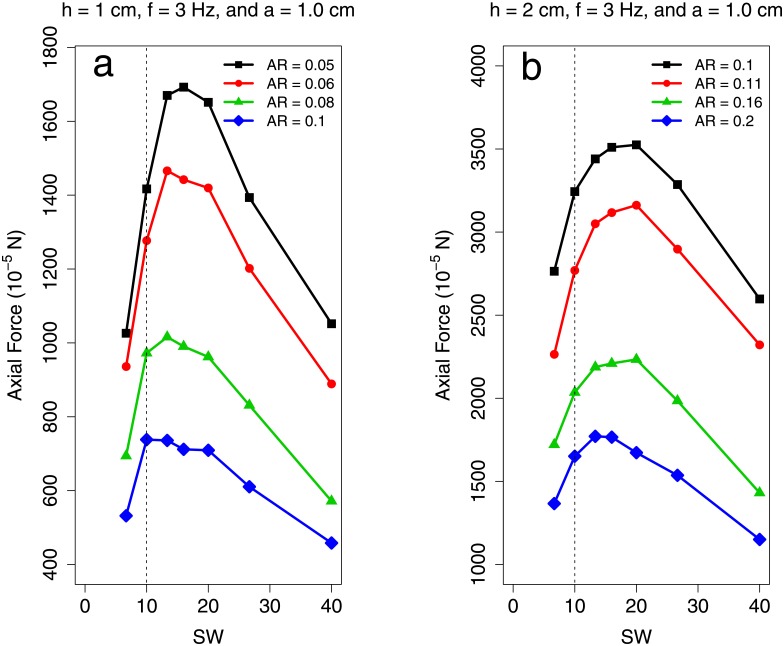
Measured propulsive force from high Re_lat_ undulating sheet simulations. The axial propulsive force generated by a stationary undulating sheet plotted against specific wavelength. In both cases (a) & (b), plate length was varied. These represent cases where Re_lat_ > 1 × 10^2^. Data are available in S2 Data.

Fig 5 shows the results of simulations done on sheets with Re_lat_ = 8.43 × 10^−1^ and Re_lat_ = 4.49. In Fig 5a & 5b, the optimal specific wavelength increases from 10 to 20 as the plate aspect ratio increases from 0.1 to 0.4 and 0.05 to 0.6 respectively. Fig 6 shows the result from simulations carried out on sheets with Re_lat_ = 3.37 × 10^2^. For these higher Reynolds number sheets, the OSW is nearly independent of AR. This indicates that there is a parametric dependence of OSW on AR for low Re_lat_ regimes that does not persist for moderate Re_lat_ swimmers. Due to computational limitations, we were unable to interrogate whether or not this aspect ratio independence continues for even higher Reynolds numbers. However for moderate Reynolds numbers, this is consistent with what is observed in the meta–analysis data: both low AR anguilliform and high AR non–anguilliform swimmers undulated at SW near 10. To our knowledge, there is no experimental observation on BCF fish with both Re_lat_ < 10^2^ and AR > 0.15.

**Fig 7:**
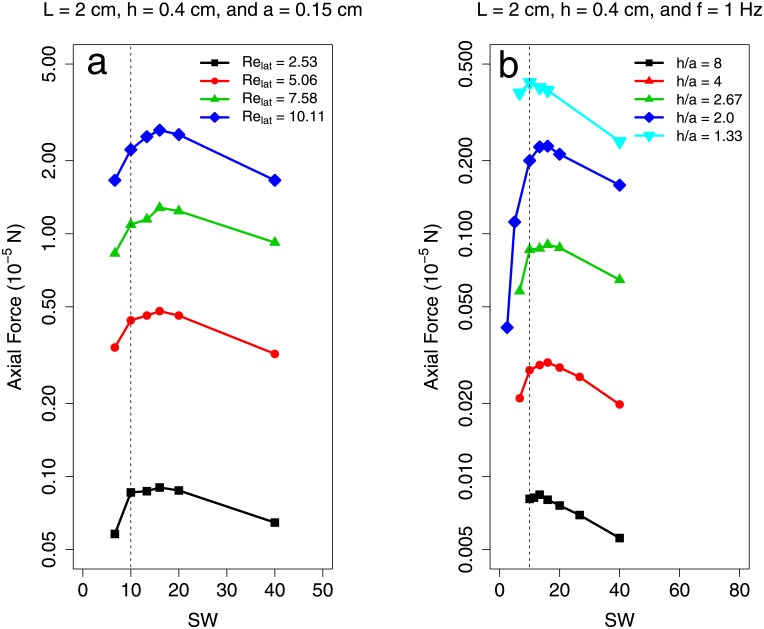
Measured propulsive force from low Re_lat_ sheets with varying frequency and amplitude. Axial propulsive force vs. specific wavelength for additional small–sheet simulations in which (a) frequency and (b) amplitude were varied. Data are available in S2 Data.

**Fig 8:**
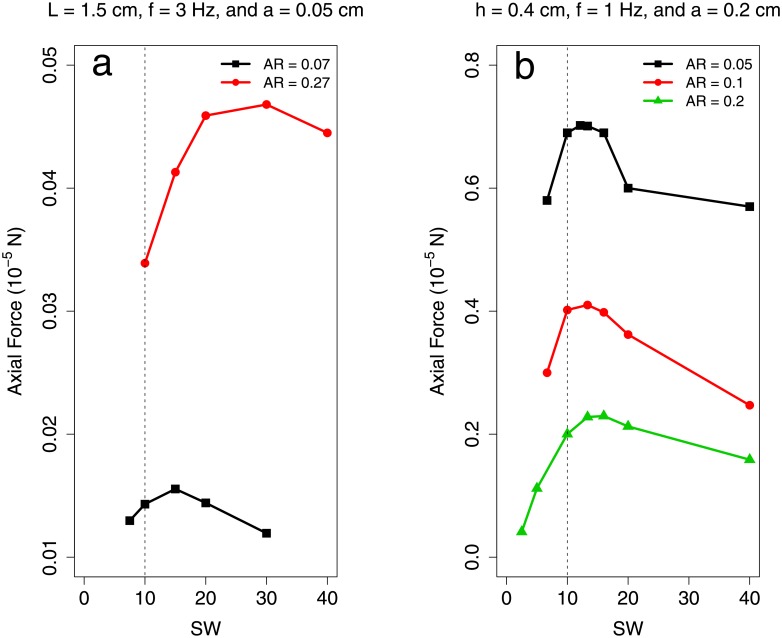
Measured propulsive force from low Re_lat_ sheets with varying span and length. Axial propulsive force vs. specific wavelength for additional small–sheet simulations with varying aspect ratios. In case (a), plate span was varied while in case (b), plate length was varied. Data are available in S2 Data.

Figs 7 & 8 show additional small–sheet simulations in which aspect ratio, Re_lat_, and amplitude were varied systematically. These show a consistent dependence of OSW on AR for low Re_lat_ and that OSW does not vary considerably as a function of frequency or amplitude. In particular, Fig 7b shows that the OSW does not vary considerably as a function of *h*/*a* for fixed AR. The range of *h*/*a* values considered match those of the observed swimmers in this study (1.15 − 8.22).

**Fig 9:**
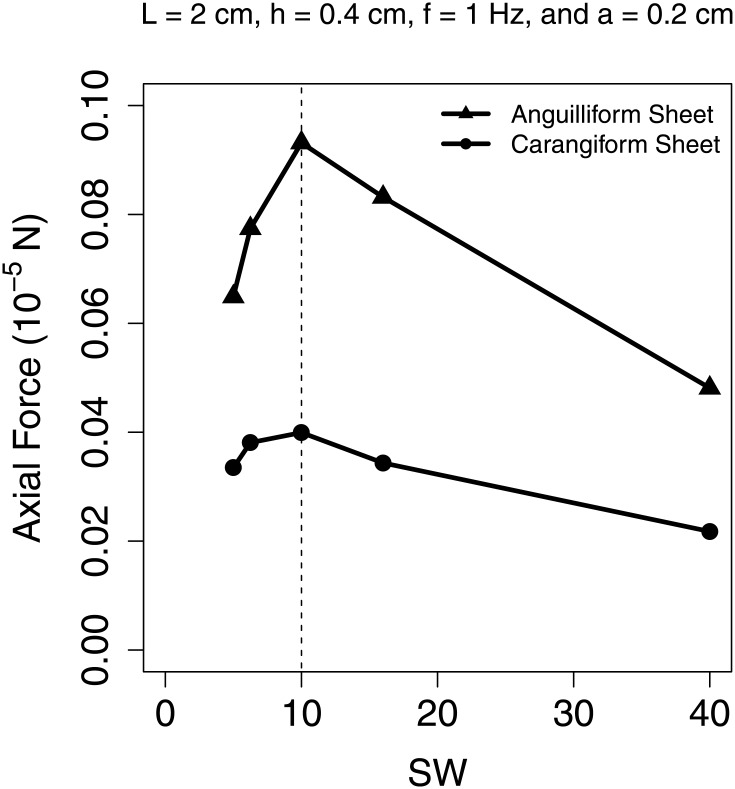
Measured propulsive force from anguilliform and carangiform sheet simulations. Axial propulsive force vs. specific wavelength for additional small–sheet simulations with prescribed anguilliform (▲) and carangiform (⚫) amplitude profiles. Data are available in S2 Data.

Fig 9 shows small–sheet simulations in which the amplitude profile *A*(*x*) was allowed to vary along its body length. Two different profiles were considered; *A*(*x*) = *ae*^*x*/*L*−1^, which represents a modeled anguilliform swimmer, and *A*(*x*) = *a*(0.2 − 0.8*x*/*L* + 1.6(*x*/*L*)^2^), which represents a modeled carangiform swimmer [16, 17]. These simulations were carried out at Re_lat_ = 4.49. We again see that propulsive force is maximized at SW = 10 in both cases, implying that the OSW criterion is valid for variable amplitude sheet swimmers.

**Fig 10:**
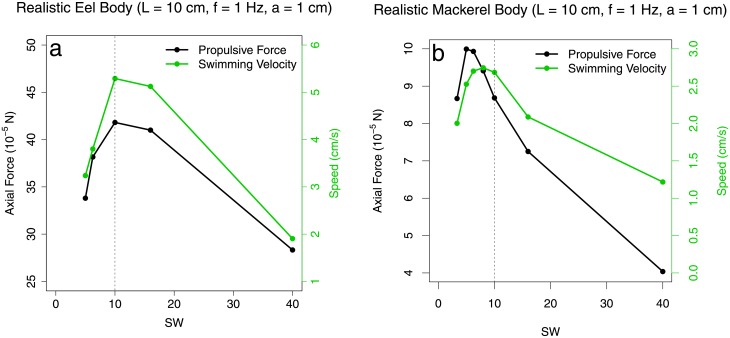
Measured swimming speed and force from realistic eel and mackerel simulations. Axial swimming speed and propulsive force computed from free–swimming (green) and translation–locked (black) simulations of undulating (a) eel bodies, and (b) mackerel bodies, plotted against the specific wavelength. In free–swimming simulations, the forward swimming speed of the undulating body was an output parameter of the simulation. These simulations were carried out at Re_lat_ = 1.12 × 10^2^, with corresponding swimming–speed Reynolds number range 1.378 × 10^3^ < Re < 5.95 × 10^3^. Data are available in S2 Data.

Finally, Fig 10a and 10b show both stationary and free–swimming simulations done on realistic eel and mackerel swimmer geometries, respectively. Although these bodies were realistically shaped, they were scaled–down in size due to the limitations of our computational tool. For the eel body, an anguilliform amplitude profile was prescribed, while for the mackerel body a carangiform amplitude profile was prescribed. These simulations were carried out at Re_lat_ = 1.12 × 10^2^, with corresponding swimming–speed Reynolds number range 1.378 × 10^3^ < Re < 5.95 × 10^3^. In all cases, there is an optimal specific wavelength that maximizes axial force and swimming speed. This implies that the OSW design principle holds for more realistic swimming bodies. Visualizations of the body geometries and simulations are provided in the Materials and Methods section.

The axial force simulation data shown in Figs 5, 6, 7, 8 & 9 were non–dimensionalized and are presented in section 2 of S1 Appendix in S2 Fig–S6 Fig.

### Robotic sheets

In [11] and [12] the authors performed a parametric study on robotic undulating plates with constant amplitude along their length and span. Although these fins were actuated from a base, these experiments resemble the simulations done on undulating rectangular sheets in this work insofar as their amplitude did not vary along their span. In these experiments, four identical free–swimming robotic plates were fully submerged in water and undulated at $f=\frac{6}{9},\;\frac{7}{9},\;\frac{8}{9},\;\text{and}\;\frac{9}{9}\;\text{Hz}$; for a given frequency, wavelength was varied in order to find the optimal λ that maximized swimming speed *U*. The plates had length *L* = 63 cm and span *h* = 20 cm, corresponding to an aspect ratio of AR = 0.32. The sheet had amplitude *a* = 5 cm and all of the experiments were carried out at Re_lat_ ∈ [1.87 × 10^3^, 2.50 × 10^3^], with corresponding swimming–speed Reynolds number range Re ∈ [5.96 × 10^4^, 1.53 × 10^5^].

The OSWs for these four cases are shown in Fig 3 alongside the OSWs from the simulations done in this study. The optimal specific wavelengths for these robotic sheets were between 10 and 13, which are consistent with simulation and animal data. Furthermore, the Re_lat_ values for these robotic sheets were higher than those used in our numerical simulations. This is evidence that SW maximizes swimming speed for high Reynolds number animals.

## Discussion

Through analysis of undulatory swimmer data, we have shown that BCF swimmers undulate at a relatively narrow range of specific wavelength values. The range of specific wavelength values observed in 27 species of steady–swimming BCF swimmers was 4.02 − 14.93 with $\bar{\text{SW}}=9.91$ and *s*_SW_ = 3.45. Additionally we observed, across a large range of Reynolds numbers, that low aspect ratio anguilliform swimmers tend to swim at greater St than high aspect ratio non–anguilliform swimmers. This is consistent with numerical studies relating the emergence of single and double–row wakes to optimal Strouhal numbers for carangiform and anguilliform swimmers, respectively [16, 17].

Using numerical simulations and data from robotic undulating sheets, we showed that the optimal specific wavelength for maximal thrust generation falls in the range 5 − 30 for undulating bodies, which closely matches the range of SW values found in observations of BCF fish. However, the morphology of the undulating sheet seemed to cause variation in the location of the OSW. Our undulating sheet simulations were a simplified model problem for actual BCF swimmers. These simulations did not capture the effect of varying span of a swimmer, which is a common trait of BCF swimmers, nor the effect of a distinct caudal fin. However, they provide a physical basis for why these swimmers might undulate within a range of SW values: the larger thrust and swimming speed attained at the OSW are advantageous. Future studies should carry out simulations with more realistic gaits in order to quantify how the OSW changes for more realistic swimming bodies. Similarly, robotic sheet data corroborate the existence of an OSW for undulatory swimmers at high Reynolds numbers, but parametric studies on more realistic BCF robots should be conducted.

To the best of our knowledge, propulsive wavelength of body/caudal fin swimmers is rarely measured in experimental studies. This work establishes the importance of λ as a kinematic parameter for propulsive performance. All of the organisms considered in this study, except for the axolotl, are body/caudal fin fish, and therefore have the same ancestry. The hope is that this work inspires more data on wavelength to be measured across a wider variety of swimming animals to determine whether this SW trait is universal among aquatic locomotors.

The optimal specific wavelength rule represents a small subset of a larger and complicated design space with various optimality principles relating the input parameters shown in Eq 2 to other cost of transport or efficiency metrics. We do not claim that the SW constraint is more important than any other optimality principle; rather, the entire landscape needs to be explored in order to determine the most optimal swimmer. However, swimming at the OSW to maximize *U*_*x*_ can be done in both low and high efficiency swimming situations. For example, consider a swimmer of fixed dimensions required to cruise a long distance at a low cost of transport. In this case, slow and steady swimming at a low frequency and amplitude is desirable. However, swimming at the OSW will maximize speed within this high efficiency regime. Conversely in the case where a swimmer needs to escape from a predator, the efficiency at which it swims is less important as it undulates its body with the highest frequency and amplitude possible. Once these maximum *f* and *a* values are achieved, swimming at the OSW would further maximize speed within this low efficiency regime. The situation can be reversed as well: for a fixed swimming body required to swim at some desired speed *U*, a SW value can be chosen to achieve this speed while the remaining parameters can be chosen to maintain this speed at a maximum efficiency.

When considering the kinematics of underwater vehicles, the specific wavelength is a quantity that can be prescribed before swimming starts while the Strouhal number is unknown until the vehicle has reached a steady–swimming speed. This is advantageous from a design standpoint as net axial force (or swimming speed) can be predicted and maximized prior to building a vehicle. However, our numerical simulations establish an OSW range for maximized thrust without considering the power spent to attain that propulsive force. Moreover, the Strouhal number has been shown to maximize propulsive efficiency, the ratio of net thrust times a desired speed to power spent [6]. Given a set of mechanical constraints on a vehicle (an immersed body of fixed *L*, *h*, and *a* that must swim at *U*), SW and St could provide an optimal choice of λ and *f*. Therefore, the Strouhal number and specific wavelength form a pair of complementary design rules. This landscape relating propulsive efficiency and thrust to SW and St will be the subject of future investigation.

Additional constraints on the kinematics of undulatory swimmers must be met when considering design criteria for underwater vehicles. It has been shown through direct numerical simulation of turbulent flow over an undulating wall that the wave–speed *V*_*f*_ = *λf* must exceed the external flow velocity *U*_0_ for thrust to be produced and turbulence to be reduced [18]. For free–swimming bodies, this forces the swimming speed *U* to be less than *V*_*f*_, which is observed for our free–swimming simulation and all the swimmers considered in this study.

Our simulations also showed that the OSW increases from 10 to 20 as the undulating plate’s aspect ratio increased from 0.05 to 0.6 when Re_lat_ < 100. This implies that a skinny swimmer needs more spatial undulations alongs its body to maximize thrust than a wide swimmer in the viscous regime. We hypothesize that the mechanisms described in out prior work [7] might explain why this occurs. In section 3 of S1 Appendix, we mathematically describe and discuss the plausibility of the two competing mechanisms. A skinny plate with small span *h* (low aspect ratio) has a disadvantage in transporting fluid efficiently when compared to a wide, high aspect ratio plate. Therefore, the low AR plate requires more waves along its body (a lower SW) to generate maximal thrust. At higher values of Re_lat_, we hypothesize that the increased length scale diminishes the importance of varying span. This parametric dependence of the OSW on aspect ratio and Reynolds number has yet to be observed for free–swimming bodies. However, consider a free–swimming fish that is undulating its body at an optimal specific wavelength value that maximizes its swimming speed, keeping all other independent parameters fixed. Now imagine this swimmer’s translational degrees of freedom are locked and the fish is constrained to remain stationary while still undulating its body with the same free–swimming deformation kinematics. We hypothesize that the net axial force generated by this stationary fish would be maximum at similar free–swimming optimal specific wavelength value, keeping all other independent parameters fixed. Simulations carried for an undulating sheets, mackerels, and eels corroborate this hypothesis, although there does seem to be some variation in OSW values between the stationary and free–swimming cases.

**Fig 11:**
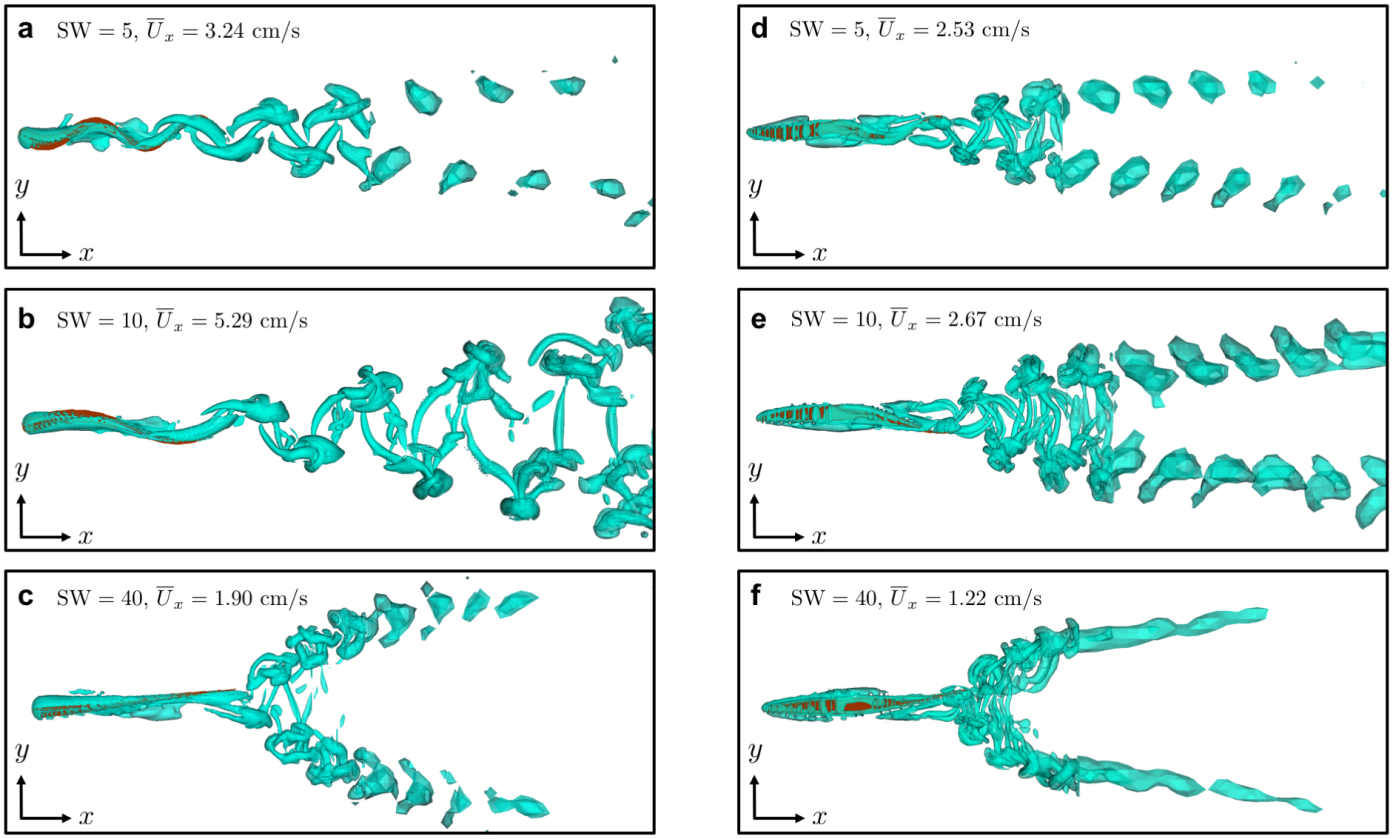
Vortical structures shed from free–swimming eel and mackerel. Three-dimensional vortical structures visualized for free–swimming simulations of an eel (a–c) and mackerel (d–f) at three different SW values. The wakes are visualized using isosurfaces of q–criterion, where $q=\frac{1}{2}\;\left(\|A{\|}^{2}-\|S{\|}^{2}\right)$, where *A* and *S* are the antisymmetric and symmetric parts of the fluid velocity gradient tensor ∇**u**, respectively.

The three–dimensional wake structures from undulating bodies has been analyzed as a function of Strouhal number in previous simulation studies [16, 17, 19]. Wake visualizations at three different SW values from the free–swimming, realistic eel and mackerel simulations conducted in this study are shown in Fig 11. The speed at which the top and bottom row wakes push away from each other seems to increase as SW increases. Additionally, it appears that the vortical structures shed from each swimmer are more organized and remain more coherent at the SW value maximizing swimming speed. Therefore, it seems likely that the types of vortices shed at the optimal specific wavelength is beneficial in some way to propulsive performance when compared to non–optimal SW values. The anatomy of these wakes should be studied in future numerical and experimental work.

Additionally, future parametric studies should further consider the effect of different amplitude and span profiles along the length of the swimming body: would a high AR swimmer with anguilliform amplitude profile maximize thrust or swimming speed at the same SW as a low AR swimmer with carangiform amplitude profile? These types of simulations were carried out in [19]; for a given body (with either low or high AR) it was found that anguilliform kinematics produce faster swimming speeds than carangiform kinematics, which is consistent with the simulations carried out in this work. Additionally, for a given undulation profile *A*(*x*), the higher AR body produced a faster swimming speed or thrust [19], which is also shown in our sheet simulations. However, the location of the OSW changes with respect to AR for low Reynolds number sheets; whether this parametric dependence holds for more realistic swimming bodies is yet to be explored.

For the BCF swimmers considered in this study, there is an adherence to a SW ∼ 10 constraint and numerical simulations suggest a possible reason for the emergence of this rule: specific wavelength maximizes the thrust or swimming speed generated by an undulatory swimmer. Similarly, it is hypothesized that the St ∼ 0.3 constraint found in flying and swimming animals emerged because the Strouhal number is a maximizer of propulsive efficiency. Consistency of two more measurements, the flexion ratio (FR) and maximum flexion angle (*θ*_*f*_), has been observed among propulsors of multiple taxonomic groups, length scales, and fluid media [20]. These quantities describe the extent of bending in non–anguilliform fish that primarily undulate their caudal peduncle and fin. These bending characteristics are important to consider because they encode information about the amplitude profile along the length of a swimming body in a dimensionless way. Although we did conduct variable amplitude profile simulations in this study, we did not study the parametric effect of FR or *θ*_*f*_ on swimming speed or efficiency. We hypothesize that these parameters also induce an optimality principle. This is evidenced by studies showing the effect of variable stiffness profiles and bending ratios on propulsive performance [21, 22]. Future work should explore these universal bending patterns for all BCF swimmers and their relationship to propulsive performance, St, and SW in order to unify various design rules for human–engineered, underwater propulsors. Doing so would specify the λ, *f* and *A*(*x*) needed to build a hydrodynamically optimal swimming machine.

## Materials and methods

### Experimental data

Analysis was done on kinematic data for 26 species of steady–swimming undulatory fish, and 1 species of salamander from a meta–analysis study [4]. Table 1 contains relevant data for the swimmers considered in the present study. These data were selected for analysis because a well–defined wavelength, frequency, amplitude, and swimming speed of the animals were reported.

**Table 1:** Mean specific wavelength, lateral Reynolds number, and aspect ratio for the organisms studied in this work. *Anguilliform swimmers. ^†^Swimmer has a distinct caudal fin. All *Clupea harengus* and *Danio rerio* specimens were larval. Aspect ratio data are measured from side–view images or schematics of swimmer and not related to the listed *N*_*i*_ value.

Species	*N*_*i*_	${\bar{\text{SW}}}_{i}\pm \text{one}\;\text{s}.\text{d}.$	$\bar{{\text{Re}}_{\text{lat}}}\pm \text{one}\;\text{s}.\text{d}.$	AR	Source
*Abramis brama* (Cyprinidae)^†^	1	7.09	1.75 × 10^3^	0.242	[4]
*Ambystoma mexicanum* (Ambystomatidae)*^†^	8	5.87 ± 1.06	1.41 × 10^3^ ± 6.86 × 10^2^	0.126	[4, 13]
Ambystoma mexicanum young*	3	4.34 ± 1.19	3.62 × 10^2^ ± 3.05 × 10^2^	0.102	[4, 14]
*Ammodytes marinus* (Ammodytidae)*^†^	4	8.16 ± 0.40	4.48 × 10^2^ ± 1.78 × 10^2^	0.056	[4]
*Anguilla anguilla* (Anguillidae)*	4	8.26 ± 1.16	3.33 × 10^2^ ± 2.34 × 10^2^	0.065	[4, 23]
*Anguilla rostrata* (Anguillidae)*	1	8.72	6.42 × 10^2^	0.068	[4, 24]
*Carassius auratus* (Cyprinidae)^†^	1	8.58	1.38 × 10^3^	0.254	[4]
*Chelon labrosus risso* (Mugilidae)^†^	2	13.96 ± 2.31	3.75 × 10^2^ ± 1.67 × 10^2^	0.186	[4, 25]
*Clupea harengus* (Clupeidae)*^†^	6	4.02 ± 0.06	2.55 × 10^1^ ± 1.69 × 10^1^	0.114	[4, 26, 27]
*Danio rerio* (Cyprinidae)*	11	5.36 ± 1.29	5.34 × 10^1^ ± 3.43 × 10^1^	0.115	[4, 5, 28]
*Esox (hybrid)* (Esocidae)^†^	1	14.92	4.43 × 10^2^	0.140	[4, 29]
*Euthynnus affinis* (Scombridae)^†^	4	14.82 ± 1.11	9.31 × 10^2^ ± 3.82 × 10^2^	0.175	[4, 30]
*Gadus morhua* (Gadidae)^†^	6	11.59 ± 2.28	2.39 × 10^3^ ± 1.74 × 10^3^	0.169	[4, 31]
*Gambusia affinis* (Poeciliidae)^†^	6	7.50 ± 1.94	1.24 × 10^2^ ± 3.95 × 10^1^	0.200	[4, 32]
*Hyperoplus lanceolata* (Ammodytidae)*^†^	5	10.09 ± 2.08	1.71 × 10^3^ ± 9.22 × 10^2^	0.077	[4]
*Lepisosteus osseus* (Lepisosteidae)*^†^	7	11.89 ± 0.90	1.99 × 10^3^ ± 6.50 × 10^2^	0.064	[4, 33]
*Leuciscus leuciscus* (Cyprinidae)^†^	1	6.03	3.08 × 10^3^	0.176	[4]
*Liza ramada* (Mugilidae)^†^	1	11.40	3.94 × 10^3^	0.168	[4]
*Micropterus salmoides* (Centrarchidae)^†^	5	12.06 ± 0.87	7.33 × 10^2^ ± 2.85 × 10^2^	0.222	[4, 34]
*Oncorhynchus mykiss* (Salmonidae)^†^	10	10.52 ± 1.19	8.18 × 10^2^ ± 3.24 × 10^2^	0.200	[4, 29]
*Pelamis platurus* (Hydrophiidae)*	2	4.14 ± 0.34	5.57 × 10^3^ ± 2.26 × 10^3^	0.070	[4, 35, 36]
*Pleuronectes platessa* (Pleuronectidae)^†^	1	13.12	5.00 × 10^2^	0.347	[4, 31]
*Pollachius virens* (Gadidae)^†^	9	11.16 ± 1.25	3.17 × 10^3^ ± 1.25 × 10^3^	0.179	[4, 37]
*Salmo salar* (Salmonidae)^†^	3	14.86 ± 5.02	4.35 × 10^3^ ± 2.65 × 10^2^	0.138	[4]
*Sarda chiliensis chiliensis* (Scombridae)^†^	2	13.43 ± 2.35	5.00 × 10^3^ ± 3.57 × 10^3^	0.151	[4, 38]
*Scomber japonicus* (Scombridae)^†^	12	12.55 ± 2.21	1.50 × 10^3^ ± 9.38 × 10^2^	0.143	[4, 30]
*Scomber scombrus* (Scombridae)^†^	9	9.46 ± 0.83	8.55 × 10^3^ ± 4.45 × 10^3^	0.134	[4, 37]
*Strongylura marina* (Belonidae)*^†^	3	13.50 ± 3.02	5.26 × 10^2^ ± 5.48 × 10^2^	0.050	[4, 39]

Data on aspect ratio (AR) for each of the species were also measured from schematics provided by the sources listed in Table 1, when available, or side–view images from http://www.fishbase.org/. Data on Re = *ρUL*/*μ* were provided for each specimen in [4]. However, for a more direct comparison between observations and simulations, the data were reinterpreted in terms of Re_lat_. We found that these two dimensionless quantities are strongly monotonically correlated with Spearman’s rank correlation coefficient *r*_*s*_ = 0.940 (see section 1 of S1 Appendix).

### Numerical simulations

The three-dimensional numerical simulations of the undulating sheet were carried out using the constraint–based immersed boundary method (cIB) developed within the IBAMR software [40, 41]. IBAMR is an immersed boundary (IB) method implementation with support for adaptive mesh refinement (AMR) and distributed memory parallelism [42]. The cIB method involves solving the Navier–Stokes momentum and mass conservation equations for a combined fluid and solid domain. The sheet kinematics and motion are represented in a Lagrangian frame and are treated as a constraint force in the momentum equation. The fluid motion is solved for on an Eulerian grid with no–slip or periodic boundary conditions used on all faces of the computational domain. We found that the choice of boundary conditions on the faces of the computational domain did not affect the thrust computation on the body of the swimmer given that the swimmer was far enough away from simulation boundaries. The body’s lateral undulations was given by a traveling wave *y*(*x*, *t*) = *A*(*x*) sin(2*π*(*x*/λ − *ft*)), where the amplitude profile is either constant (*A*(*x*) = *a*) or variable (*A*(*x*) = *ae*^*x*/*L*−1^ for anguilliform swimmers and *A*(*x*) = *a*(0.2 − 0.8*x*/*L* + 1.6(*x*/*L*)^2^) for carangiform swimmers. No–slip boundary conditions were enforced on the surface of the swimmer.

**Fig 12:**
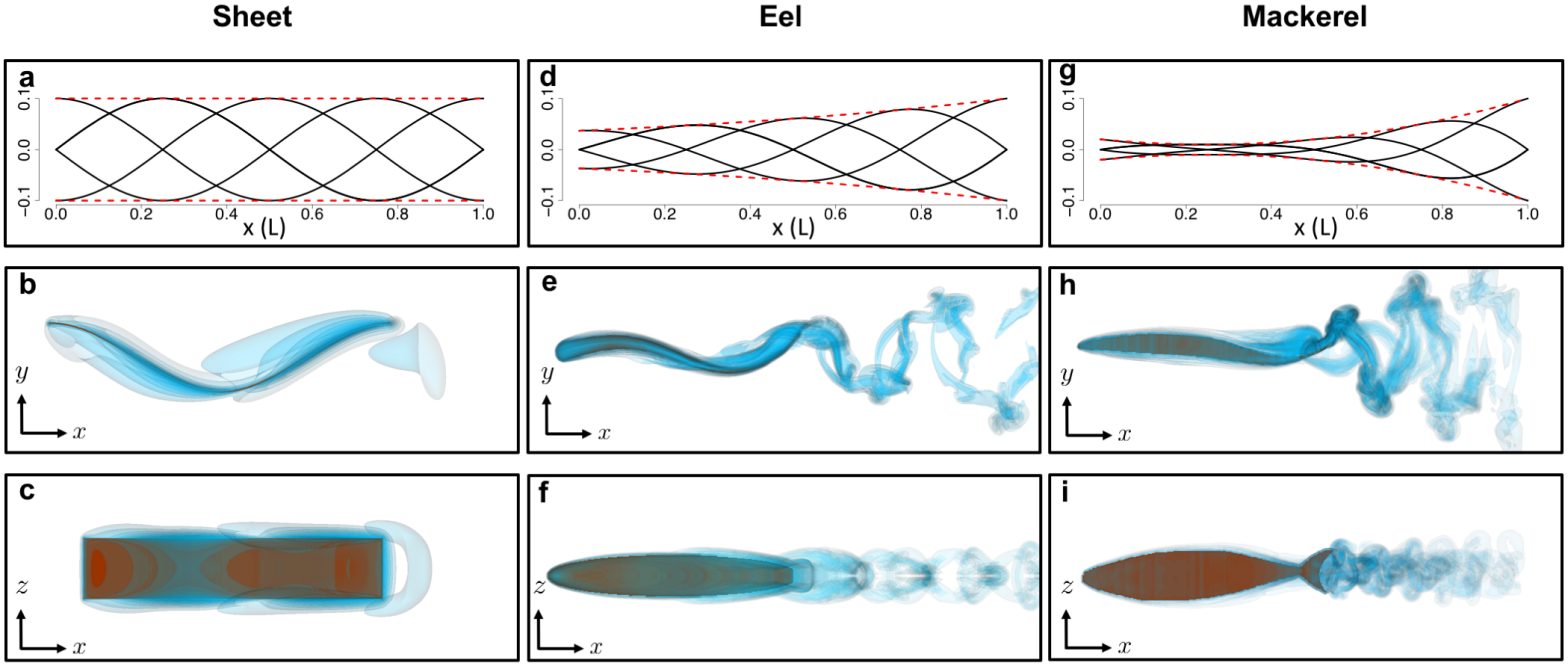
Wake and body visualizations from free–swimming simulations. Top figures show the midline kinematics (black) over time for the three different types of undulating bodies considered in the present numerical study. Dashed red line denote the amplitude function ±*a*(*x*). Middle and bottom figures show contours of vorticity magnitude for the three bodies. Middle figures show the top–view with undulations present in the lateral direction, while bottom figures show the side–view of each body. (a), (b), & (c) An undulating flat plate with SW = 10 and Re_lat_ = 4.49; the low Reynolds number causes the wake to remain large and mostly attached. (d), (e) & (f) An undulating eel body with SW = 10 and Re_lat_ = 1.12 × 10^2^. (g), (h), & (i) An undulating mackerel body with SW = 10 and Re_lat_ = 1.12 × 10^2^.

Three different undulating bodies were considered in this study: sheets, eels, and mackerels. Top–and side–view visualizations from free–swimming simulations are shown in Fig 12. Simulations were carried out for a wide range of morphological and kinematic parameters as described in the text. The fluid had physical properties corresponding to water at 25°C with density *ρ* = 1 g/cm^3^ and viscosity *μ* = 0.89 × 10^−2^ g/(cm ⋅ s).

A grid–convergence test was conducted for every set of simulations in order to validate the numerical technique. Let Δ**X** = (Δ*x*, Δ*y*, Δ*z*) be the grid–spacing at the finest mesh level and *q* be the desired measured quantity (i.e. axial force or swimming speed). Simulations were conducted at an initial grid–spacing Δ**X**_0_ with measured quantity *q*_0_, and a refined grid–spacing Δ**X**_1_ = Δ**X**_0_/2 with measured quantity *q*_1_. If the percent change *δ* = |*q*_1_ − *q*_0_|/*q*_0_ × 100% between these two measurements was less than 10%, then the rest of the simulations in that set were conducted at the coarser grid–spacing Δ**X**_0_. Otherwise, we ran a more refined case at Δ**X**_2_ = Δ**X**_0_/4 and compared *q*_1_ and *q*_2_. This refinement continued until a *δ* < 10% was achieved and a grid–spacing was decided. This process was done every time Re_lat_ changed by an order of magnitude. All simulations were carried out at a finest grid–spacing (5.86, 4.81, 4.46) × 10^−3^ cm ≤ Δ**X** ≤ (6.25, 6.25, 6.25) × 10^−2^ cm and time–step 1 × 10^−4^ s ≤ Δ*t* ≤ 5 × 10^−4^ s. A similar grid–refinement study was conducted in [16] to validate the drag measurements on a mackerel swimmer at Re = 4000.

For free–swimming simulations, the various Reynolds numbers ranges were as follows: Re ∈ [2.25 × 10^2^, 5.95 × 10^3^], Re_lat_ ∈ [4.49 × 10^0^, 1.12 × 10^2^], Re_lat,*L*_ ∈ [4.49 × 10^1^, 1.12 × 10^3^], and Re_wave_ ∈ [2.25 × 10^2^, 4.49 × 10^4^]. For translation–locked simulations, the various Reynolds numbers ranges were as follows: Re = 0, Re_lat_ ∈ [2.81 × 10^−1^, 3.37 × 10^2^], Re_lat,*L*_ ∈ [1.12 × 10^1^, 6.74 × 10^3^], and Re_wave_ ∈ [4.21 × 10^1^, 2.70 × 10^5^]. Since the Reynold numbers considered were relatively modest (except for Re_wave_, which is generally much larger than Re during free–swimming; see section 1 of S1 Appendix) and the high velocities were confined to the region close to the plate, no turbulence model was used in the present study. All the simulations were conducted with zero incoming velocity. Visualizations from the simulations were checked to ensure that the domain sizes were sufficiently large to minimize the interaction between the walls of the computational domain and the immersed body.

**Fig 13:**
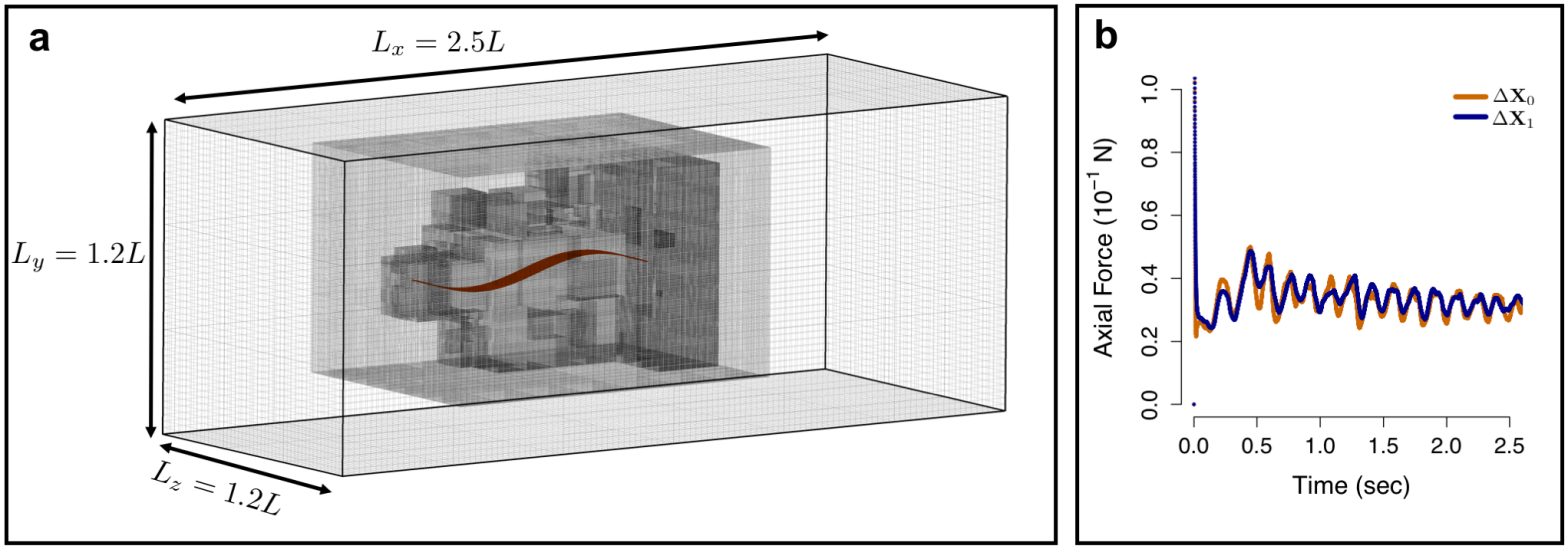
Computational setup and grid refinement validation case for an undulating sheet. (a) Computational setup for a translation–locked simulation of an undulating sheet with *L* = 20 cm, *h* = 2 cm, *f* = 3 Hz, *a* = 1 cm and λ = 20 cm, which corresponds to Re_lat_ = 3.37 × 10^2^. Three adaptive mesh levels are shown and the immersed structure is always placed on the finest mesh level. (b) Time evolution of axial force generated by the sheet for two different grid spacing values: Δ**X**_0_ = (3.125, 3.125, 3.125) × 10^−3^ *L* (orange) and Δ**X**_1_ = (1.5625, 1.5625, 1.5625) × 10^−3^ *L* (blue).

Fig 13 shows an example of the computational setup and grid–refinement validation for a translation–locked sheet undulating at Re_lat_ = 3.37 × 10^2^ and SW = 20. For the coarser grid simulation, Δ**X**_0_ = (3.125, 3.125, 3.125) × 10^−3^ *L*, while for the refined grid simulation, Δ**X**_1_ = (1.5625, 1.5625, 1.5625) × 10^−3^ *L*. The mean axial force measurements for each case are *q*_0_ = 33.6 mN and *q*_1_ = 34.3 mN, which represents a percent change of *δ* = 2.08%. This gives us confidence that the high Re_lat_ simulations carried out in this work are relatively insensitive to grid spacing up to the prescribed *δ* tolerance.

## Supporting information

S1 Appendix Supporting appendix. Section 1 shows the correlation between swimming–speed, wave–speed, and lateral Reynolds numbers. Section 2 discusses the nondimensionalization of the axial force simulation data included in Figs 5–9. Section 3 describes how to quantify the mechanisms underlying the OSW result.
(PDF)

S1 Fig Correlation between different Reynolds numbers. The swimming–speed Reynolds number Re vs. Re_lat_ (⚫; blue), Re_lat,*L*_ (♦; purple), and Re_wave_ (◼; red) for the swimmers considered in [4], along with the lines of best fit. The best fit lines are given by log Re = 1.803 + 1.340log Re_lat_, log Re = −0.4058 + 1.2306log Re_lat,*L*_, and log Re = −1.450 + 1.083log Re_wave_.
(EPS)

S2 FigDimensionless axial force data from Fig 5.Dimensionless axial propulsive force $\hat{F}$ generated by a stationary undulating sheet plotted against specific wavelength. In both cases (a) & (b), plate span was varied. These data represent cases where (a) Re_lat_ = 8.43 × 10^−1^ and (b) Re_lat_ = 4.49.(EPS)

S3 FigDimensionless axial force data from Fig 6.Dimensionless axial propulsive force $\hat{F}$ generated by a stationary undulating sheet plotted against specific wavelength. In both cases (a) & (b), plate length was varied. These data represent cases where Re_lat_ = 3.37 × 10^2^. Data are available in S2 Data.(EPS)

S4 FigDimensionless axial force data from Fig 7.Dimensionless axial propulsive force $\hat{F}$ vs. specific wavelength for additional small–sheet simulations in which (a) frequency and (b) amplitude were varied. These data represent cases where (a) 2.53 ≤ Re_lat_ ≤ 10.11 and (b) 0.28 ≤ Re_lat_ ≤ 10.16. Data are available in S2 Data.(EPS)

S5 FigDimensionless axial force data from Fig 8.Dimensionless axial propulsive force $\hat{F}$ vs. specific wavelength for additional small–sheet simulations with varying aspect ratios. In case (a), plate span was varied while in case (b), plate length was varied. These data represent cases where (a) Re_lat_ = 8.43 × 10^−2^ and (b) Re_lat_ = 4.49. Data are available in S2 Data.(EPS)

S6 FigDimensionless axial force data from Fig 9.Dimensionless axial propulsive force $\hat{F}$ generated by a stationary undulating sheet with prescribed anguilliform (▲) and carangiform (⚫) amplitude profiles plotted against specific wavelength. These data represent cases where Re_lat_ = 4.49. Data are available in S2 Data.(EPS)

S7 FigMomentum transfer by an undulatory swimmer.At the front of the swimmer, the body sucks stationary fluid with negligible momentum *m*_in_ and accelerates it downstream. This ejected fluid is often manifested as a wake with momentum $m_{\text{wake}}∼{ℳ}_{\text{wave}}(\lambda f)$. Finally, this wake eventually dissipates further downstream as it loses momentum.(TIFF)

S8 Fig Correlation between maximum axial fluid velocity and traveling wave velocity. The maximum fluid axial velocity *u*_max_ generated by the sheet over Ω and over one swimming cycle [*t*_0_, *t*_0_ + *T*] vs. the traveling wave speed *λf*. Data are available in S3 Data.
(EPS)

S9 Fig Quantitative analysis of the velocity and friction mechanisms. (*a*) Net axial force generated by simulations of an undulating plate with *L* = 1.0 cm, *h* = 0.1 cm, *f* = 3 Hz, *a* = 0.05 with (red) periodic boundary conditions, and (black) wall boundary conditions. Average axial fluid momentum multiplied by undulation frequency over one swimming cycle with periodic boundary conditions (green) as functions of SW. (*b*) velocity of the traveling wave *λf* (pink) and change in weighted mass (blue). Data are available in S3 Data.
(EPS)

S10 Fig Qualitative analysis of the velocity mechanism. Mid–sheet (*z* = −0.05 cm) contours of axial fluid velocity for a stationary sheet simulation for various snapshots in time. Black dots represent the Lagrangian points of the undulating body. The plate has *L* = 1.0 cm, *h* = 0.1 cm, *f* = 3 Hz, *a* = 0.05 cm with (a) & (b) SW = 13.33, (c) & (d) SW = 10, (e) & (f) SW = 5.
(EPS)

S11 Fig Qualitative analysis of the friction mechanism. Mid–sheet (*z* = −0.05 cm) contours of normalized axial fluid velocity (*u*_*x*_/*u*_max_) for a stationary sheet simulation for various snapshots in time. Black dots represent the Lagrangian points of the undulating body. The plate has *L* = 1.0 cm, *h* = 0.1 cm, *f* = 3 Hz, *a* = 0.05 cm with (a) & (b) SW = 13.33, (c) & (d) SW = 10, (e) & (f) SW = 5. For each difference SW, *u*_max_ is taken to be the maximum axial fluid velocity over Ω and over one swimming cycle.
(EPS)

S1 Data Observational and robotic data plotted in Figs 1–3. (XLS)

S2 Data Data plotted in Figs 4–10 and S2 Fig–S6 Fig. (XLS)

S3 Data Data plotted in S8 Fig and S9 Fig. (XLS)
