# Supplementary material for: Optimal specific wavelength for maximum thrust production in undulatory propulsion
Source: PLoS One. 2017 Jun 27;12(6):e0179727. doi: 10.1371/journal.pone.0179727 (PMC5487070; doi:10.1371/journal.pone.0179727)
Supplement: S1 Appendix — Section 1 shows the correlation between swimming–speed, wave–speed, and lateral Reynolds numbers. Section 2 discusses the nondimensionalization of the axial force simulation data included in Figs 5–9. Section 3 describes how to quantify the mechanisms underlying the OSW result. (PDF) [file pone.0179727.s001.pdf]

Supporting Appendix: Optimal Specific Wavelength for Maximum Thrust Production in Undulatory Propulsion

Nishant Nangia[1], Rahul Bale[2], Nelson Chen[2], Yohanna Hanna[2] and Neelesh A. Patankar[1,2,*]

[1]Engineering Sciences and Applied Mathematics, Northwestern University, Evanston, IL, USA
[2]Department of Mechanical Engineering, Northwestern University, Evanston, IL, USA
*Author for correspondence (n–patankar@northwestern.edu)

1 Correlation Between Various Reynolds Numbers

The Reynolds number $\mathrm{Re} = \rho U_x L/\mu$, where U_x is the forward swimming speed of the animal, is generally used to categorize the ratio of inertial to viscous forces in free–swimming [1]. However for the translation–locked simulations carried out in this study, this conventional Reynolds number is always zero and does not provide any useful information about the flow regime. In the main text, we present a nondimensionalization of the axial force generated by a translation–locked undulating fin (repeated here in Eqs. S1 and S2) and a dimensionless quantity called the lateral Reynolds number $\mathrm{Re}_{\mathrm{lat}} = \rho(fa)a/\mu$ is obtained. We note in the main text that a variety of Reynolds numbers could replace $\mathrm{Re}_{\mathrm{lat}}$ in the nondimensionalization, including the wave–speed Reynolds number $\mathrm{Re}_{\mathrm{wave}} = \rho(\lambda f)L/\mu$ or $\mathrm{Re}_{\mathrm{lat},L} = \rho(fa)L/\mu$.

The Spearman's rank correlation coefficient $r_s(X,Y)$ measures how well the relationship between two quantities can be described by a monotonic function. A r_s value close to 1 indicates that X and Y increase nearly in tandem. In Fig S1, we show that all these dimensionless quantities correlate strongly with Re for the swimming animals considered in [1]. For these data, $r_s(\mathrm{Re}_{\mathrm{lat}}, \mathrm{Re}) = 0.940$, $r_s(\mathrm{Re}_{\mathrm{lat},L}, \mathrm{Re}) = 0.974$, and $r_s(\mathrm{Re}_{\mathrm{wave}}, \mathrm{Re}) = 0.988$. Hence, our analysis is independent of the chosen dimensionless Reynolds number. We chose to analyze the data with respect to $\mathrm{Re}_{\mathrm{lat}}$ because it is independent of wavelength.

2 Parametric Study: Dimensionless Force Results

For a translation–locked sheet of length L and span h, the force generated by the undulations depends on the physical input parameters of the system:

$$F_x = fn(\rho, \mu, f, \lambda, L, h, a), \tag{1}$$

in which ρ and μ are the density and viscosity of the fluid respectively, f is the frequency of the sheet, λ is the wavelength, and a is the (constant) amplitude of the undulation. In the main text, the following nondimensionalization of Eq. S1 is given

$$\widehat{F} = \frac{F_x}{\frac{1}{2}\rho(fa)^2 Lh} = fn\left(\frac{\lambda}{a}, \frac{\rho(fa)a}{\mu}, \frac{h}{L}, \frac{h}{a}\right) = fn\left(\mathrm{SW}, \mathrm{Re}_{\mathrm{lat}}, \mathrm{AR}, \frac{h}{a}\right). \tag{2}$$

In the main text, we present axial force data vs. specific wavelength from a numerical parametric study of sinusoidally undulating sheets (see Figs 5 – 9). In Figs S2 – S6, we show the dimensionless force $\widehat{F}$ data from these same simulations. In all of the simulations considered in this study, the OSW that maximizes F_x and $\widehat{F}$ are the same.

3 Quantifying the Friction and Velocity Mechanisms

Let an undulating sheet (or fish body) of length L, span h, constant amplitude a, frequency f and wavelength λ be immersed in a fluid with density ρ and velocity $\vec{u}(x, y, z, t) = (u_x, u_y, u_z)$. Let Ω be the domain containing the sheet. Consider that the sheet is undulating but not translating and that there is no externally imposed flow on the sheet.

A stationary undulating sheet sucks stationary fluid from the front end and accelerates it to some momentum at the downstream end. This is applicable at all finite Reynolds number flows in this work. The fluid momentum ejected at the downstream end is usually manifested in the form of a wake, which is prominent at high Reynolds numbers. The acceleration of the fluid from front to back is caused by a backwards force from the sheet on the fluid, which by Newton's third law is equal to the forward thrust force from the fluid on the sheet (Fig S6). The momentum in the wake is eventually dissipated/decelerated further downstream (Fig S6). This dissipation corresponds to a net force on the walls of the domain, which, under "steady" conditions, is equal and opposite to the thrust force on the sheet. This can be formally shown by applying momentum conservation to appropriate control volumes. Consequently, the forward (thrust) force F_x on a stationary undulatory body can be estimated by [2, 3, 4, 5, 6]

$$F_x \sim \mathcal{M}_{\mathrm{wake}}(\lambda f) f, \tag{3}$$

in which $\mathcal{M}_{\mathrm{wake}}$ is a measure of the fluid mass mobilized by the sheet (also added mass in reactive theory for thrust generation [5]) or ejected into the wake, λf is the wave velocity of undulation, and f is the frequency of undulation.

In an earlier work [7] and in this work we find that F_x is maximized at some optimal specific wavelength (OSW) of undulation. It was hypothesized in the earlier work [7] that two competing mechanisms cause thrust to be maximized at the optimal specific wavelength. A high wavelength λ leads to a higher wave velocity ($= \lambda f$) and fluid is pushed backward faster at a given frequency; this was called the velocity mechanism. However, since the waves are "shallower" at high wavelength, fluid is transported less efficiently. Conversely, a low λ leads to "deeper" waves and thus fluid is transported backwards more efficiently, but

it is done so at a slower speed; this was called the friction mechanism. In the context of Eq. S3, $\mathcal{M}_{\text{wake}}$ would quantify the friction mechanism and λf would quantify the velocity mechanism. To interrogate the plausibility of the hypothesis for OSW proposed in [7], each of the quantities F_x, $\mathcal{M}_{\text{wake}}$, and λf need to be quantified independently.

The thrust F_x is computed directly by integrating forces on the sheet and λf is known based on chosen parameters. In Fig S7, we show that the maximum fluid axial velocity $u_{\max}$ generated by the sheet over a single swimming cycle is directly correlated to the traveling wave velocity λf. Therefore, the velocity mechanism is quantified by wave speed.

It is not straightforward to compute $\mathcal{M}_{\text{wake}}$, which quantifies how much fluid is mobilized and accelerated by the force F_x exerted by the sheet on the fluid. Note that downstream from the wake the fluid is in fact decelerated, which corresponds largely to forces on the wall. In general, the accelerating and decelerating domains of the fluid surrounding the sheet are not clearly separated.

In order to overcome the above issue, we choose to consider a different but related problem. We perform undulating sheet simulations in a fully periodic domain – no wall boundaries are included. In this case, the force from the sheet on the fluid causes the fluid in the entire domain to be accelerated through one period of undulation – the fluid does not decelerate downstream from the sheet because there is no resistance from the walls.

The total momentum of the fluid in the computational domain is given component–wise as

$$(m_x, m_y, m_z) = \int_{\Omega} \rho \vec{u}\, dV. \tag{4}$$

We choose to analyze a sheet with $L = 1.0$ cm, $h = 0.1$ cm, $f = 3$ Hz, $a = 0.05$ cm with three different SW values: 5, 10, and 13.33. For this sheet, axial thrust is maximized at SW = 10. The sheet occupies the area $[0, 1] \times [-0.1, 0]$ in the xz-plane with minimum and maximum extents in the undulation direction at $y = -0.05$ cm and $y = 0.05$ cm. For these simulations, $\Omega = [-1.7, 4.3] \times [-1.1, 0.9] \times [-1.2, 0.8]$ with periodic boundary conditions on all faces, unless otherwise stated.

In our simulated sheet cases, we compute the average thrust force over a full swimming cycle $[t_0, t_0 + T]$, starting at some time t_0 after an oscillatory steady–state has been reached in the axial force. Here, $T = 1/f$ is the sheet period. In Fig S8a, we show F_x directly computed by integrating forces on the sheet. We show F_x computed on the sheet in a fully periodic domain and for another case where there are walls present. All other parameters, including domain size are the same. Both forces are identical. As desired, the periodic boundary condition did not affect thrust production.

The average axial force should satisfy the following equation,

$$F_x = f \cdot [m_x (t_0 + T) - m_x(t_0)] = f \cdot \Delta m_x, \tag{5}$$

where $m_x(t)$ is in general nonzero at t_0. Fig S8a also plots $f\Delta m_x$ in a periodic domain with $f = 3$ Hz for all cases. The comparison between F_x and $f\Delta m_x$ is consistent with Eq. S5; the error is attributed to numerical integration.

Now, we argue that the two aforementioned mechanisms (velocity and friction) can be defined by analyzing the change in fluid momentum in Eq. S5. Note that although a large λ sheet may generate high velocities, the volume over which these high velocities are realized

also contributes to the momentum (through integration over volume elements dV in Eq.(S4)). There are no clearly separated domains of mobile or immobile fluid. Therefore, we choose to quantify the amount of fluid being transported by using a weight factor $u_x/\lambda f$ over the entire volume. Mathematically, this is equivalent to splitting m_x into

$$m_x = \lambda f \int_\Omega \rho \frac{u_x}{\lambda f}\, dV = \lambda f \cdot \mathcal{M}, \tag{6}$$

where $\mathcal{M}$ is measured in unit of mass. The integral term equal to $\mathcal{M}(t)$ represents the amount of mobilized fluid weighted by $u_x/\lambda f$; this quantifies how much fluid is transported at axial velocities close to the theoretical maximum. Using Eq. S5, the average thrust force on the sheet is given by

$$F_x = f \cdot \lambda f \left(\mathcal{M}(t_0 + T) - \mathcal{M}(t_0)\right) = f \cdot \lambda f \cdot \Delta\mathcal{M}, \tag{7}$$

where λf and $\Delta\mathcal{M}$ are, by definition, quantitative measures of the velocity and friction mechanisms, respectively. Eq. S7 is analogous to Eq. S3. $\Delta\mathcal{M}$ quantifies how much additional fluid is mobilized by force F_x over one periodic interval of duration T. Quantitatively, the competing mechanisms are shown in Fig S8b. As SW increases, λf increases, while $\Delta\mathcal{M}$ decreases, as hypothesized.

Figs S9 and S10 show velocity contours for the cases considered. In Fig S9, contours of velocity in the immediate vicinity of the sheet are plotted for three different SW values for two snapshots in time. Notice that as SW decreases, the maximum velocity also decreases (also plotted in Fig S7). However in Fig S10, we show the same instances in time, but instead plot contours of $u_x/u_{\max}$. We choose to normalize by the maximum fluid axial velocity $u_{\max}$ generated by the sheet over Ω and over one swimming cycle $[t_0, t_0 + T]$ to illustrate the differences between these sheets. Now as SW decreases, the area of influence of the maximum velocities increases i.e. there are more areas of dark red in the SW = 5 case than in the SW = 13.33 case.

References

[1] van Weerden, J. F., Reid, D. A. P. & Hemelrijk, C. K. A meta-analysis of steady undulatory swimming. *Fish and Fisheries* **15**, 397–409 (2014).

[2] Dickinson, MH & Götz, KG The wake dynamics and flight forces of the fruit fly Drosophila melanogaster. *The Journal of Experimental Biology* **199**: 2085–2104 (1996).

[3] Drucker, EG & Lauder, GV Locomotor forces on a swimming fish: three-dimensional vortex wake dynamics quantified using digital particle image velocimetry. *The Journal of Experimental Biology* **202**: 2393–2412 (1999).

[4] Lighthill, MJ Aquatic animal propulsion of high hydromechanical efficiency. *Journal of Fluid Mechanics* **44**: 265–301 (1970).

[5] Lighthill, MJ Large-Amplitude Elongated–Body Theory of Fish Locomotion. *Proceedings of the Royal Society of London* **179**: 125–138 (1971).

[6] Gazzola, M, Argentina, M. & Mahadevan, L Scaling macroscopic aquatic locomotion. *Nature Physics* **10**: 758–761 (2014).

[7] Bale, R, Neveln, ID, Bhalla, APS, MacIver, MA & Patankar, NA Convergent evolution of mechanically optimal locomotion in aquatic invertebrates and vertebrates. *PLOS Biology* **13**: e1002123 (2015).
