# Supplementary figures and images for: Optimal specific wavelength for maximum thrust production in undulatory propulsion
Source: PLoS One. 2017 Jun 27;12(6):e0179727. doi: 10.1371/journal.pone.0179727 (PMC5487070; doi:10.1371/journal.pone.0179727)

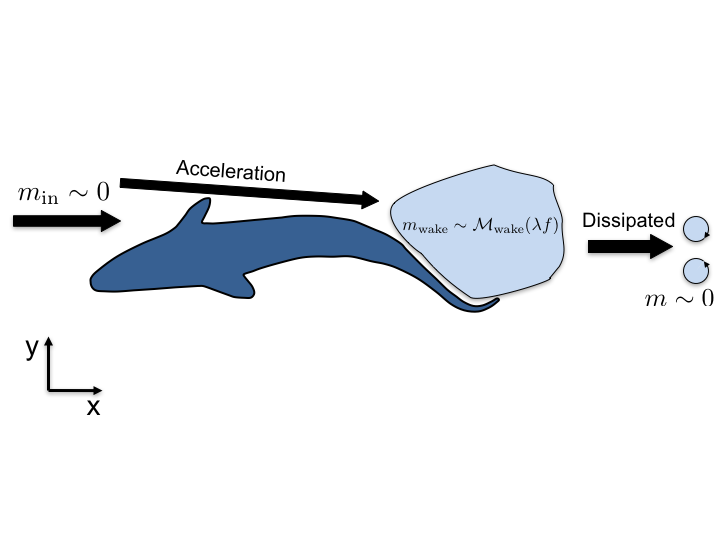

Supplement: S7 Fig — At the front of the swimmer, the body sucks stationary fluid with negligible momentum min and accelerates it downstream. This ejected fluid is often manifested as a wake with momentum mwake∼ℳwave(λf). Finally, this wake eventually dissipates further downstream as it loses momentum. (TIFF) [file pone.0179727.s008.tiff]
